# Paternal Effects in Mammalian Reproduction: Functional, Environmental, and Clinical Relevance of Sperm Components in Early Embryos and Beyond

**DOI:** 10.1002/mrd.70020

**Published:** 2025-03-23

**Authors:** Keith E. Latham

**Affiliations:** ^1^ Department of Animal Science Michigan State University East Lansing Michigan USA; ^2^ Department of Obstetrics, Gynecology and Reproductive Biology Michigan State University East Lansing Michigan USA

**Keywords:** epigenetic, microRNA, paternal factor, semen, sperm quality, sperm transcriptome, transgenerational effects

## Abstract

In addition to widely recognized contributions of the paternal genome, centriole, and oocyte‐activation factors, sperm deliver a wide range of macromolecules to the fertilized embryo. The impacts of these factors on the embryo, progeny, and even subsequent generations have become increasingly apparent, along with an understanding of an extensive potential for male health and environmental exposures to exert both immediate and long‐term impacts on mammalian reproduction. Available data reveal that sperm factors interact with and regulate the actions of oocyte factors as well as exerting additional direct effects on the early embryo. This review provides a summary of the nature and mechanisms of paternal effects in early mammalian embryos, long‐term effects in progeny, susceptibility of sperm components to diverse environmental factors, and potential approaches to mitigate adverse effects of such exposures.

## 1 Introduction

Parental effects on embryo development and progeny phenotype are widespread across diverse phylogenetic groups. Maternal effects, wherein oocyte components exert extensive effects on such processes as gene regulation, embryo physiology, axial patterning, and cell fate specification, are mechanistically well‐characterized (Ruebel and Latham 2020), although in mammals, certain mechanisms of maternal effects play lesser roles than in other animal taxa. The mechanisms of paternal effects are less well‐characterized, given the lesser contribution of cellular materials to the zygote. However, recent studies have provided significant new insights into paternal effect mechanisms and increased awareness of the interactions between sperm‐derived and oocyte‐derived factors.

**Figure 1:**
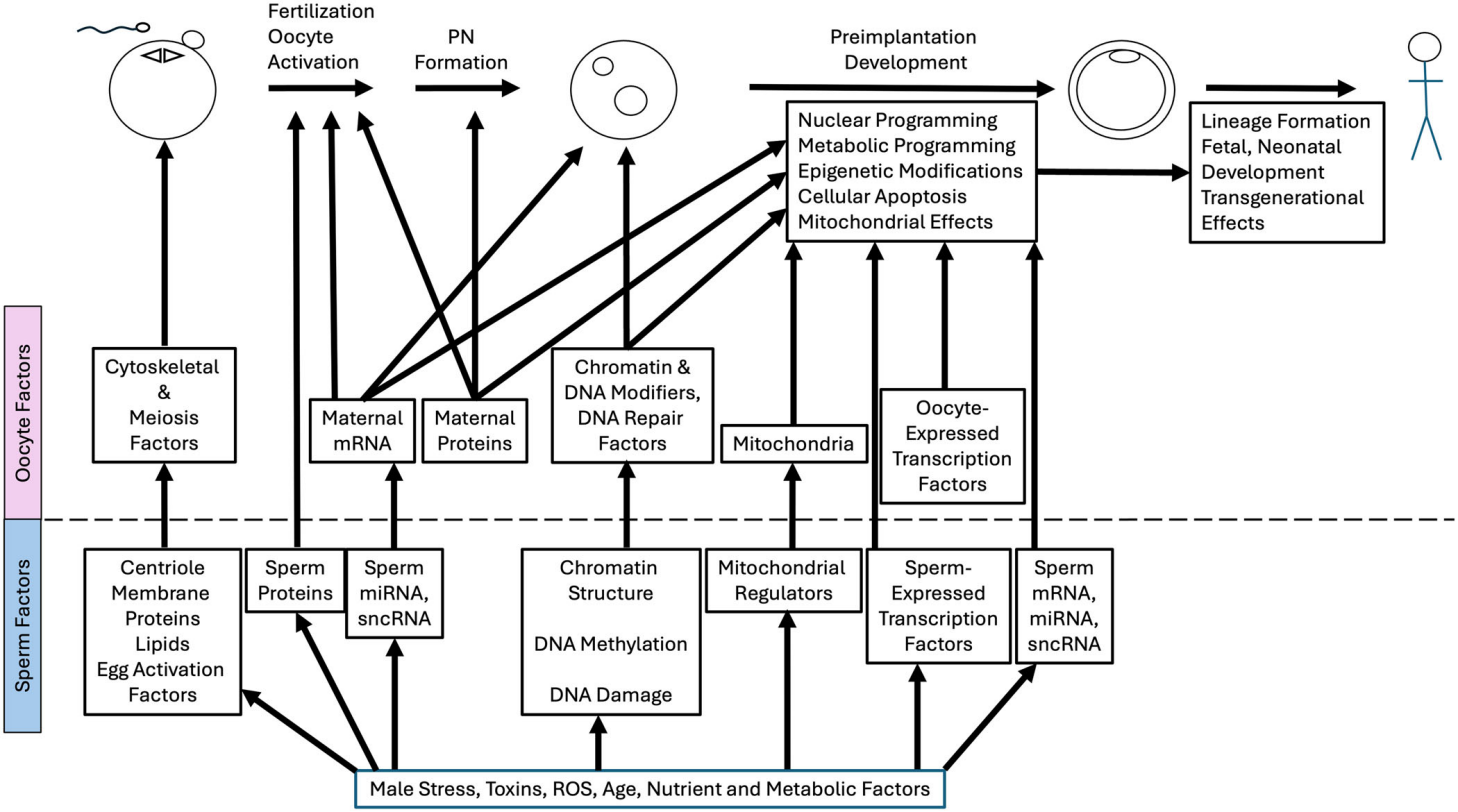
Summary of connections between paternal (sperm) and maternal (oocyte) factors and essential processes during embryogenesis and long‐term impacts on progeny. Sperm factors are below the dotted line, and oocyte factors are above the dotted line. Note that many sperm factors mediate effects via impacts on oocyte factors, whereas other sperm factors may exert more direct effects on some processes. Some sperm factors may exert effects via multiple pathways. Environmental factors such as stress, diet, obesity, and toxin exposures can exert additional paternal effects via sperm components. Cellular stages depicted are, from left to right, gametes (sperm and metaphase II stage oocyte), zygote, blastocyst, and progeny. ROS, reactive oxygen species.

Previously recognized essential sperm contributions to the embryo included a highly compacted haploid genome, centrioles, and factors that trigger oocyte activation (Tarozzi et al. 2021). More recently, mRNA, microRNAs (miRNAs), DNA‐bound transcription and chromatin regulatory factors, other sperm‐borne proteins, membranes and lipids, DNA damage, and the sperm epigenome have emerged as other key factors having potential roles in development and in determining embryo phenotype (Lockhart et al. 2023; Tarozzi et al. 2021) (Figure 1). Many of these sperm components may be impacted by exogenous environmental influences such as heat, male age, male diet, stress and toxins, any of which can have immediate as well as long‐term effects on progeny, and even transgenerational effects (Ashapkin et al. 2023; Celeghini et al. 2024; Cescon et al. 2020; Korolenko and Skinner 2024; Lismer and Kimmins 2023). This review summarizes these newer discoveries as well as prior observations to provide a comprehensive understanding of the nature and mechanisms of paternal effects in early mammalian embryos, long‐term effects in progeny, susceptibility of relevant sperm components to diverse environmental factors, and potential approaches to mitigating adverse effects of such exposures.

### 1.1 Sperm Genome Integrity

The delivery of a healthy, intact paternal genome to the oocyte is key to the fertilization process. However, sperm genome integrity can be compromised at multiple steps during spermatogenesis, spermiogenesis within the testis, and during post‐testicular stages as sperm are transported along the epididymis (Alvarez et al. 2023). Both single‐stranded and double‐stranded DNA breaks can arise, with the double‐stranded breaks being less numerous but of greater concern for their risk to embryo health (Alvarez et al. 2023). Double‐stranded DNA breaks can reside in sperm due to a failure to be repaired during mitosis, failure to be repaired during meiosis, inability to remove breaks arising through topoisomerase activity during protamine packaging, and oxidative damage due to free radical exposure in the epididymis, with opportunities to repair these breaks limited or absent during post‐meiotic and post‐testicular stages (Alvarez et al. 2023). Consequently, the capacities of the oocyte and cleaving embryo to repair these DNA breaks is crucial, but these capacities may be exceeded even in high‐quality oocytes and embryos, and can be even more problematic in oocytes or embryos of lesser quality or in oocytes subjected to cryopreservation (Khajedehi et al. 2024). It was proposed that a threshold of DNA strand breaks may be reached at which significant deficiencies in embryo development emerge due to a burden of unrepaired DNA breaks (Alvarez et al. 2023).

Male age impacts sperm genome integrity and associated fertility (Kaltsas et al. 2024). Men over the age of 50 can display increased rates of sperm DNA fragmentation, reduced semen quality parameters, and reduced ability of semen to yield live births following assisted reproduction procedures (Kaltsas et al. 2024; Morris et al. 2021). However, the effect of male age may be sensitive to other variables. One study reported no effect of male age when using fresh embryo transfer (Punjani et al. 2022). Other studies reported significant interactions with maternal age, with male age effect being more noticeable with female age over 25, and sperm DNA fragmentation increasing with male age (Marsidi et al. 2021). These observations on male age effects echo the dependence of the effects of sperm DNA damage on the oocyte ability to repair DNA damage after fertilization.

Lipids can positively or negatively affect sperm quality, fertilization rate, and even embryo phenotype, depending on the specific lipid studied (Kalo et al. 2022; Yuan et al. 2023). Such effects can relate in part to multiple processes, such as mitochondrial function, sperm motility, effects on sperm cryopreservation outcome, sperm‐egg fusion and membrane function, and membrane signaling capacities in the embryo, some of which can subsequently affect the embryo (Al‐Ali et al. 2024; Dalal et al. 2020; Hernandez‐Falco et al. 2022; Lamas‐Toranzo et al. 2020; Quinn et al. 1980; Watson 1981). However, male dietary lipids, lipids in semen extenders in vitro, and lipids employed during sperm cryopreservation can exert effects on the embryo via sperm plasma and mitochondrial membrane changes, affecting the incidence of DNA damage in sperm (Ezz et al. 2017).

Interestingly, there are conflicting results in the literature about the magnitude of effect of sperm DNA breaks on embryo viability and subsequent development. Some studies have reported significant impacts of sperm DNA fragmentation on various measures of embryo quality, embryo development and pregnancy outcomes (Gao et al. 2023; Ribas‐Maynou et al. 2022), but other studies observed limited or no correlations with embryo developmental potential (AmirJannati et al. 2024; Broussard et al. 2023; K. Liu et al. 2023; Rios et al. 2021). Such discrepancies may reflect inter‐study variability in source of sperm (i.e., testicular vs. epididymal), age and health of sperm and oocyte donors, and quality of oocytes employed (i.e., donors vs. patients), mechanisms of fertilization employed (i.e., IVF vs. intracytoplasmic sperm injection [ICSI]), and the criteria employed to surveil DNA damage and to define high versus low sperm DNA fragmentation Index. Another study found that DNA fragmentation in sperm used for ICSI in mice led to short‐term effects on preimplantation development and offspring number, as well as long‐term effects including tumors, increased postnatal weight gain, behavioral effects such as increased anxiety and memory defects, premature aging, and shortened lifespan; these effects were proposed to be due to incomplete DNA repair in the embryo (Fernandez‐Gonzalez et al. 2008). Further study, and comparisons between study outcomes may facilitate refinements in analytical and diagnostic procedures to detect and quantify sperm DNA damage, establishing operational thresholds for selection of preferred approaches to fertility treatment in individual patients, such as the use of testicular versus ejaculated sperm preparations (Alvarez et al. 2023; AmirJannati et al. 2024; K. Liu et al. 2023), and improving the ability to mitigate paternal age effects on the prevalence of DNA breaks in sperm (Rios et al. 2021).

Overall, it appears that genetic factors in males affecting the occurrence, prevalence and rate of DNA strand break repair, genetic factors in females affecting oocyte and embryo abilities to repair DNA damage, other factors affecting oocyte DNA repair capacity (e.g., age), and environmental factors impacting rates of DNA damage in sperm could all positively or negatively affect the overall level of paternal genome integrity after fertilization and the impact of paternal genome integrity on embryo phenotype. Moreover, the embryo has a remarkable ability to overcome the presence of DNA damage, with genetically mosaic embryos that have a significant burden of aneuploid cells nevertheless being able to develop into healthy progeny (Latham 2024). Thus, the paternal effect of sperm DNA breaks on the embryo is likely both quantitative and variable due to multiple interacting maternal, paternal and environmental factors.

### 1.2 Sperm Epigenome Integrity

Epigenetic features of sperm genomes could affect gene expression in the early embryo. Sperm epigenetic modifications are associated with male fertility (Leggio et al. 2024). Two main categories of epigenetic features to be considered here are sperm chromatin proteins and sperm DNA methylation.

Sperm chromatin becomes highly condensed by being repackaged with protamines. Correct protamination allows faithful transmission of an intact sperm genome to the embryo, facilitates correct chromatin remodeling and paternal pronucleus formation after fertilization, and contributes to embryo development (Okada and Yamaguchi 2017; Ribas‐Maynou et al. 2023). However, the sperm genome is not fully protaminated. Histone modifications, histone variants, and sperm‐specific transition proteins play key roles in repackaging the sperm DNA onto protamines (Lismer and Kimmins 2023). Mouse and human sperm genomes retain up to 1% and 15% histone content, respectively, and these histones are both evolutionarily conserved and associated with important regulatory genes (Lismer and Kimmins 2023). Correct sperm genome protamination not only facilitates packaging and delivery of the paternal genome to the oocyte, but it also contributes to successful post‐fertilization repackaging of the paternal genome with histones, and to the correct activation of embryonic gene transcription (Lismer and Kimmins 2023). Moreover, modifications of sperm histones and other proteins can affect how the maternal chromatin modification machinery interacts with the paternal genome, and can affect progeny health (Lismer and Kimmins 2023; Okada and Yamaguchi 2017; van de Werken et al. 2014) (Figure 1).

The histones that remain associated with sperm chromatin after protamination include different variants that are differentially methylated and differentially associated with specific regions in the sperm genome, and that display evolutionary conservation with specific epigenetic marks. These regions correspond to genes that are transcriptionally regulated during spermatogenesis or transcriptionally repressed in the early embryo (Erkek et al. 2013; Lambrot et al. 2021; Lismer and Kimmins 2023). Many histones that are retained on the sperm DNA remain associated with the paternal genome after pronucleus formation (Brykczynska et al. 2010), and thus could affect gene regulation in the embryo.

Chromatin immunoprecipitation – DNA sequencing (ChIP‐seq) of human sperm DNA targeting histone H3 lysine 4 trimethylation (H3K4me3), a marker of active gene transcription, identified large regions of associated DNA with enrichment at the transcription start site, 5′‐untranslated region, first exon, and promoter, as well as enrichment for unmethylated CpG rich regions of DNA (Lambrot et al. 2021). This overlap between H3K4me3 and CpG rich regions of DNA was also reported in mice (Erkek et al. 2013). H3K4me3‐bound DNA from non‐CpG islands was enriched for genes related to meiosis and spermatogenesis (Lambrot et al. 2021). H3K4me3 binding was also enriched for promoters bearing SINE elements, corresponding to genes expressed highly during spermatogenesis (Lambrot et al. 2021) and with developmental gene promoters (Hammoud et al. 2009). Additionally, H3K4me3‐bound DNA associated with low‐complexity repeat elements and SINE elements also encoded genes expressed in the early human embryo (Lambrot et al. 2021). H3K4me3‐bound DNA was predominantly hypomethylated, but a fraction was hypermethylated (Lambrot et al. 2021). Many of the latter genes showed enriched expression in human spermatids and were poorly expressed in embryos, suggesting a role for DNA methylation in preventing embryonic gene expression, but some exceptions to this trend were observed (Lambrot et al. 2021). H3K4me3‐bound DNA from hypomethylated regions was associated with early human embryo expression (Lambrot et al. 2021).

Additionally, DNA methylation in mouse sperm is associated with lack of histone retention (Erkek et al. 2013). The majority of histone H3 associated with sperm DNA in mice is the H3.3 variant associated with gene silencing in spermatids, but H3.1 and H3.2 histone variants are associated with regions of H3K27me3 and genes that are repressed in preimplantation stage embryos (Erkek et al. 2013). The sperm DNA associated H3K27me3 was also reported to mark developmental genes and genes that are repressed in mouse and human embryos (Brykczynska et al. 2010; Hammoud et al. 2009) and was restricted to CpG island‐containing promoters (Brykczynska et al. 2010). Histone H3 lysine 4 dimethylation (H3K4me2) on sperm DNA also associates with some developmental gene promoters (Hammoud et al. 2009) and with genes related to spermatogenesis and homeostasis (Brykczynska et al. 2010). Intriguingly, H3K4me2, H3K4me3 and H3K27me3 features mark overlapping but also distinct sets of genes (Brykczynska et al. 2010; Hammoud et al. 2009).

A remarkable degree of conservation of H3K4me3 and H3K27me3 sperm DNA associations was reported, including a core set of marked genes identified across five mammalian species (Lesch et al. 2016). Overall, these results reveal retention of modified histones indicative of gene activation or repression at DNA locations, promoting gene expression that supports spermatogenesis, but also repressing genes in the early embryo and providing an important mechanism whereby epigenetic programing of the paternal genome and paternal exposures (Lismer et al. 2021) can have a significant transgenerational impact on progeny phenotype.

As indicated above, many of the modified histones are associated with sperm DNA CpG islands, indicating potential interactions with DNA methylation. DNA methylation can regulate the expression of key genes during spermatogenesis, provide for genome imprinting, and repress endogenous retroviruses (review, Rotondo et al. 2021). Retention of such methylation patterns in the embryo could affect early development. Species differences are seen in how maternal and paternal gamete DNA methylation is handled in embryonic cells, but some key features are conserved (Okae et al. 2014; Smith et al. 2014). Despite genome reprogramming and extensive active paternal genome demethylation in the early embryo (Oswald et al. 2000; Santos et al. 2002), genomic imprints (review, Okada and Yamaguchi 2017) and many paternal differentially methylated regions (DMRs) containing variable number tandem repeat elements are retained after fertilization (Okae et al. 2014). Differences in sperm DNA methylation pattern between high and low fertility bulls were reported, and associated with transcriptome differences, although impacts on embryo viability were not clear (Kropp et al. 2017). Differences in paternal genome methylation comparing the inner cell mass and trophectoderm of human embryos from young versus aged fathers were reported (Denomme et al. 2024), as well as associations between sperm DNA methylation and male fertility (review, Ashapkin et al. 2023) and male age (Ashapkin et al. 2023). Age‐related changes in sperm DNA methylation can be transmitted to progeny and affect phenotype (Ashapkin et al. 2023). Beyond these observations, an extensive body of literature exists related to sperm DNA methylation defects related to impaired male fertility (review, Rotondo et al. 2021). Collectively, these data not only highlight key features of DNA methylation relative to clinical practice but also highlight the importance of the sperm epigenome as a mediator of paternal effects in embryos and progeny (Figure 1).

### 1.3 Sperm Transcriptome Contributions to the Embryo

A remarkable discovery tracing back over three decades (Gòdia et al. 2018; Goodwin 2000; Kumar et al. 1993; Miller 2000; Miller et al. 1994; Richter 1999; Rohwedder et al. 1996) is that mature ejaculated sperm possess a substantial collection of RNAs, including mRNAs detected initially by RT‐PCR and in situ hybridization, and later by microarray and RNA‐sequencing. Moreover, at least some of these sperm RNAs are transferred to the embryo upon fertilization (Ostermeier et al. 2004). Some sperm mRNAs localize to the post‐acrosomal midpiece portion of the tail (Kumar et al. 1993), but RNAs are also found in other sub‐cellular regions, including the inner and outer nucleus and the outer membrane (reviews, Gòdia et al. 2018; Santiago et al. 2021). Many sperm RNAs are fragments of protein‐coding mRNAs, but intact mRNAs are present (Santiago et al. 2021). In some early studies, short and long interspersed nuclear repetitive elements (SINEs and LINEs) were observed as major components of the sperm RNA population (Miller 2000; Miller et al. 1999). Early observations led to considerations of the possible contributions of technical assay characteristics to outcomes, versus biological relevance, such as whether the sperm mRNA encoded essential proteins required during final stages of spermiogenesis, whether post‐meiotic gene expression occurred, and whether sperm mRNAs were merely remnants of mRNAs expressed at earlier stages (Kramer 1997; Miller 2000). As more powerful methods of sperm RNA analysis became applied, the complexity of the sperm mRNA population became more evident (Santiago et al. 2021).

Studies employing whole transcriptome analyses yielded novel insights into the functional significance of sperm mRNA (Gòdia et al. 2018; Santiago et al. 2021). Confirmation that sperm mRNAs are conveyed to the embryo upon fertilization was achieved using the zona‐free hamster egg human sperm penetration assay combined with RT‐PCR (Ostermeier et al. 2004). Microarray analyses of human sperm revealed thousands of mRNAs in ejaculated sperm, and further revealed that many of these mRNAs are not present in oocytes, and some encode proteins related to embryogenesis and morphogenesis (Dadoune et al. 2005; Ostermeier et al. 2002). An RNA sequencing study of bovine sperm, oocyte and two‐ to four‐cell stage embryos revealed 472 exclusively sperm‐derived mRNAs in the embryos (Gross et al. 2019). A meta‐analysis of RNA sequencing studies from human, mouse, cow and horse sperm revealed a complex mixture of mRNAs, as well as small RNAs, long noncoding RNAs, transposable elements, intronic RNA elements and other RNA types (Jodar et al. 2013). These and many other studies have suggested the possible use of sperm RNA sequencing in evaluating male fertility, and understanding some of the mechanisms by which male fertility may be reduced by environmental, nutritional, and diverse health factors (Hernandez‐Silva et al. 2022; Jodar et al. 2015; Llavanera 2024; Mehta and Singh 2024; Santiago et al. 2021).

An additional possibility that emerged is that the sperm mRNA contributes to embryo phenotype (Gòdia et al. 2018; Rassoulzadegan et al. 2006; Santiago et al. 2021) (Figure 1). The sperm abundances of some porcine mRNAs was correlated with subsequent embryo cleavage rates (Hwang et al. 2013). An RNA sequencing study of bovine two‐ to four‐cell stage embryos revealed 65 differentially express genes comparing embryos produced using sperm from high or low fertility bulls, of which 4 were sperm‐derived (Gross et al. 2019). Absence of some human sperm mRNAs was associated with reduced live birth rates achieved via timed intercourse or intrauterine insemination, an effect that appears to be overcome using IVF or ICSI (Jodar et al. 2015). It was postulated that the abrogation of an effect on live birth rate could be due to IVF and ICSI, which may bypass defects in sperm function, and possible effects on the embryo that are overcome by selection of embryos for transfer (Jodar et al. 2015). A meta‐analysis of sperm mRNAs across different mammalian species found that some of the mRNAs encode epigenetic or chromatin modifiers, suggesting potential post‐fertilization effects on the embryo epigenome and on embryonic genome remodeling (Jodar et al. 2013). An assessment of human sperm mRNAs for correlation with male body mass index (BMI) (Tomar et al. 2024) identified transcripts related to chromosome organization, and chromatin remodeler cofactors, RNA interactors, readers, erasers, and writers (CREWS) as well as adipogenesis, stress and inflammation, leading to potential effects on sperm function and possibly the embryo (Swanson et al. 2020). Another study identified exonic RNA elements for which increased or decreased sperm abundances correlated with embryo blastocyst formation rate, and these included mRNAs related to mitosis, phosphorylation/dephosphorylation, and ectoderm and endoderm development (Hamilton et al. 2024). Knockdowns of some of these genes led to early developmental lethality (Hamilton et al. 2024).

The potential for long‐term effects of sperm mRNAs on progeny, and even transgenerational effects, was raised by observations of non‐Mendelian epigenetic effects, that is, RNA‐mediated paramutation (review, Chen, Yan, and Duan 2016). For example, injecting miRNAs into zygotes can induce heritable epigenetic changes impacting progeny growth (Grandjean et al. 2009). In one study, sperm from *Kit* mutant mouse males displayed increased RNA content and altered species of *Kit* mRNA in the testis (Rassoulzadegan et al. 2006). Microinjection of total RNA from *Kit* mutant mouse tissues into zygotes phenocopied the *Kit* mutation in progeny (Rassoulzadegan et al. 2006). The *Cdk9* mRNA is present in sperm, and injecting coding region fragments of the *Cdk9* mRNA into mouse zygotes induced heritable cardiac hypertrophy (Wagner et al. 2008).

The data obtained to date thus implicate sperm mRNAs in contributing to essential sperm functions and to essential early events in the embryo, such as chromatin remodeling, epigenetic modifications, nuclear programming, genome activation, transposable element activity (Hamilton et al. 2024), effects on primary germ layers, and potential long‐term effects on progeny phenotype via paramutation. Distinguishing between sperm and embryo functions, distinguishing between effects of mRNA and other RNA elements, distinguishing between sperm‐expressed and sperm‐extrinsic mRNAs (e.g., mRNAs acquired from exogenous sources within the epididymis such as extracellular vesicles [EVs] [Santiago et al. 2021]), defining the exact population of sperm mRNAs that enter the zygote, demonstrating embryonic protein expression from sperm‐derived mRNAs, and demonstrating subsequent impacts on embryo phenotype and relevant mechanisms of those impacts remain important objectives for better understanding the sperm mRNA functions in the early embryo (Figure 1).

Small noncoding sperm RNA (sncRNA) elements have been notably associated with embryo phenotype, with miRNAs and tRNA‐derived small RNAs (tsRNAs) being particularly striking in this respect (Chen, Yan, Duan 2016; Hamilton et al. 2024; Jodar et al. 2013; Klastrup et al. 2019; Santiago et al. 2021) (Figure 1). Sperm sncRNA content can be affected by male diet, and subsequently affect early embryo gene expression, metabolism and long‐term phenotype (Klastrup et al. 2019). Studies injecting miRNAs into zygotes yielded striking effects on progeny phenotype, phenocopying results obtained with fragmented mRNA from sperm in the *Kit* and *Cdk9* models (Rassoulzadegan et al. 2006; Wagner et al. 2008). Injecting sperm miRNAs from males subjected to various stressors can induce degradation of essential maternal mRNAs in zygotes (Rodgers et al. 2015) and alter progeny phenotype (Grandjean et al. 2015; Rodgers et al. 2015). The ability of sperm RNA to recapitulate maternal stress effects on behavior and metabolism in progeny when injected into zygotes was attributed to changes in sperm miRNA abundances experienced in those males (Gapp et al. 2014). A conserved role has been found for miRNA‐34c as a regulator of maternal mRNA degradation (Cui et al. 2023). Sperm miR‐34c regulates the *Bcl2* mRNA in mouse zygotes and it has been postulated following miR‐34c inhibitor treatment that miR‐34c thereby plays a role in regulating embryonic cleavage (Liu et al. 2012); however, parthenogenetic activation leads to cleavage, and the effect of the inhibitor was not seen in parthenotes (Liu et al. 2012), calling into question the claim of an absolute requirement of miR‐34c for cleavage. Sperm also contain a population of tsRNAs (Peng et al. 2012). Microinjection of sperm‐expressed tsRNA into zygotes can affect embryo and progeny metabolic phenotype, including alterations in the embryonic transcriptome, affecting 62 of 922 potential target mRNAs in eight‐cell stage embryos (Chen, Yan, Cao, et al. 2016). Injecting sperm tsRNA from aged males can induce anxiety‐like behavior (Guo et al. 2021). Interestingly, tsRNA content in zygotes is lower than unfertilized eggs and parthenotes, which was postulated to indicate a fertilization‐induced consumption of tsRNAs as they execute their functions (Peng et al. 2012). Small sperm‐expressed RNAs have been proposed to have high interest as markers of sperm quality (review, Mehta and Singh 2024). The abundances of specific sperm miRNAs in semen are associated with aging (Paoli et al. 2019). Effects of sncRNAs could include paramutation, effects on chromatin structure and other epigenetic effects, regulation of mRNA stability and translation, and regulation of transposable elements (Chen, Yan, and Duan 2016). The specific actions and mechanisms by which sncRNA effects are achieved remain to be discovered for most sperm‐associated sncRNAs.

Sperm RNA modifications may also impact sperm RNA actions in the embryo (Chen, Yan, and Duan 2016). Sperm tsRNA is subject to modifications in 5‐methylcytosine and 5‐methylguanine content in response to environmental factors (Chen, Yan, Cao, et al. 2016; Chen, Yan, and Duan 2016). Moreover, paramutation in response to sperm RNA requires DNA methyltransferase 2‐mediated RNA modification (Kiani et al. 2013). Such modifications can affect small RNA stability and actions (Chen, Yan, and Duan 2016).

Collectively, the data obtained for sperm mRNAs as well as sncRNAs and other RNAs point to a significant potential for paternal effects in mammalian embryos. These effects could be mediated through diverse mechanisms including protein expression from sperm mRNA, paramutation, miRNA effects on embryonic gene expression or maternal mRNA stability and translation, and effects on expression of transposable elements, to name a few (Figure 1).

### 1.4 Sperm Proteome

In addition to sperm histone proteins discussed above, sperm contain other proteins that could affect the embryo (Figure 1). These include other chromatin‐associated or DNA‐associated proteins as well as proteins in other cellular compartments. Sperm‐associated proteins can be produced endogenously during spermatogenesis and spermiogenesis, or they may be acquired exogenously from surrounding reproductive tract fluids (Castillo et al. 2018) and EVs as postulated for small RNAs (Chen, Yan, and Duan 2016). In a meta‐analysis of proteomics studies that utilized and compared proteomes from sperm oocytes and embryos, 6871 sperm proteins were identified, of which 93 have roles in early embryo development, and 560 have roles in regulating transcription, DNA methylation, histone modifications, and noncoding RNA metabolism (Castillo et al. 2018). Deficiencies in homologs of 59 of these proteins in mice are associated with significant preimplantation embryo impairment, with attendant roles in cell adhesion and cell signaling during initial cleavage divisions, at the morula stage, or during blastocyst formation (Castillo et al. 2018). Blastocyst proteomes revealed 108 proteins in blastocysts that appear to be sperm‐derived, including proteins involved in small GTPase signal transduction, epigenetic changes and DNA damage repair; further studies to definitively test origin and function of these proteins in the early embryo should be highly informative (Castillo et al. 2018). The sperm proteome meta‐analysis also revealed 165 proteins potentially acquired from reproductive tract fluid or reproductive tract EVs, with potential functions in regulating gene expression, ion transport, extracellular matrix disassembly, cell adhesion immune system processes, and cell death, among others (Castillo et al. 2018).

An analysis of protein binding sites associated with open chromatin in mouse sperm yielded CCCTC Binding Factor (CTCF) among other proteins and indicated CTCF association with mature sperm DNA (Johnson et al. 2016). It was suggested that CTCF and other bound factors may help establish higher order chromatin structure and thereby contribute to the initial gene regulation in early embryos (Johnson et al. 2016).

In addition to chromatin regulators and transcription factors, sperm proteins related to other processes also have important roles in the early embryo. Most notable among these is PLCζ1, which initiates calcium ion oscillations upon fertilization, leading to egg activation (Gupta et al. 2022; Saunders et al. 2002). The concentration of sperm PLCζ1 in the sperm and the structural stability of the sperm affect the rate of egg activation and success in ICSI, particularly in the cow, where PLCζ1 is less abundant than in human sperm; human sperm promote better activation and cleavage rates following ICSI than do bovine sperm (Alvarez et al. 2024). The pattern of calcium oscillations during oocyte activation can impact gene regulation in the embryo as well as subsequent embryo development (Choi et al. 2013; Ozil et al. 2006), and different methods of oocyte activation for in vitro embryo production are optimum for different species (Kharche and Birade 2013; Lu et al. 2018). Because of these effects of calcium oscillations during oocyte activation on the embryo, sperm PLCζ1 could exert long‐term effects on embryo viability. A proteomics study comparing sperm proteins between patients associated with positive or negative pregnancy outcomes following ICSI identified 53 proteins that were differentially expressed (Rivera‐Egea et al. 2021). These were associated with motility, anerobic metabolism, protein biosynthesis, protein folding, and signal transduction, and included some membrane proteins (Rivera‐Egea et al. 2021).

### 1.5 Sperm Centriole and Centrosomal Proteins

In non‐rodent mammals, the sperm centrosome with its proximal centriole and centrosome proteins, plays crucial roles in conjunction with essential ooplasmic centrosomal and cytoskeletal proteins in organizing the sperm aster required for syngamy, in the formation of embryonic mitotic spindles to support faithful chromosome segregation during cleavage, and in the organization of microtubules to support intracellular transport, communication and signaling, all of which contribute to developmental competence of the embryo (Bashiri et al. 2021; Hewitson et al. 2000; Hochi 2016; Schatten and Sun 2009, 2011). Studies using ICSI demonstrated intermale variations in sperm centrosome ability to direct sperm aster formation (Hewitson et al. 2000), and using ICSI to introduce human sperm into nonhuman oocytes (e.g., rabbit, cow, pig) can reveal differences in centrosome function and sperm aster‐forming ability amongst sperm of different quality (Hochi 2016). Sperm with morphological or controsomal defects are less able to form healthy sperm asters, and such defects are associated with reduced fertility (Hewitson et al. 2000; Hochi 2016; Jaiswal et al. 2022; Terada 2004). Interestingly, development can be improved by augmenting ICSI with artificial oocyte activation treatments (Hochi 2016). The sperm centriole and centrosome proteins can thus have extended paternal effects on embryo development.

### 1.6 Semen Factors

Seminal plasma (SP) is another important avenue by which paternal effects can be mediated on the embryo (Vallet‐Buisan et al. 2023). Multiple mechanisms for SP effects on the embryo exist, including effects on the production of chemokines and cytokines by the female reproductive tract, modulations of the immune system within the female reproductive tract including effects on maternal tolerance to paternal antigens, effects on uterine receptivity, trace element availability to the sperm and subsequent delivery to the embryo, and EV components such as proteins and sncRNAs that can be taken up by the sperm and affect sperm function, fertilization, and early embryogenesis (Vallet‐Buisan et al. 2023). A comparison of SP EV RNAs using semen from men of couples after successful or unsuccessful IVF attempts identified a small number of differentially expressed circular RNAs and piRNAs associated with signaling and developmental processes (Oluwayiose et al. 2023b). Another study compared sncRNA profiles of SP EVs and identified 57 RNA elements of diverse types correlated with semen quality (Oluwayiose et al. 2023a). As discussed above, differences in SP sncRNAs could affect multiple functions in the early embryo. Combining SP EV component differences with the potential indirect effects mediated via the maternal reproductive tract, these observations support the possible effect of SP on early embryogenesis, subsequent progeny development, and perhaps even later generations via transgenerational effects.

Another potential class of proteins that may be relevant to sperm biology and paternal effects is the cellular prion protein family. Proteins of this family are expressed in male and female reproductive tracts (Miranda et al. 2013), Sertoli cells (Peoc'h et al. 2002), spermatogonia, spermatocytes and spermatids (Miranda et al. 2013), and in the epididymis (Gatti et al. 2002). Prion proteins can affect sperm function and may be acquired by mature sperm, and may protect sperm against oxidative stress, thereby maintaining fertility (Behrens 2002; Miranda et al. 2013; Pereira et al. 2018). However, another study failed to observe sperm cryoprotective effects of prion proteins, indicating apparent limits on prion protein protective effects (Reiten et al. 2018). Whether transfer of prion proteins to the embryo via the sperm could also affect embryo metabolism or other functions such as pluripotency is an interesting possibility (Miranda et al. 2013).

### 1.7 Extrinsic Factors Impacting Sperm and Semen Quality

Numerous studies have revealed transgenerational effects of paternal or male‐line ancestral exposures on phenotype, including effects on human health and mortality and effects in animal models (Pembrey et al. 2014). In human studies, for example, exposures (e.g., smoking) and dietary factors during early life in males were associated with effects on cardiovascular and diabetic mortality and longevity in children or grandchildren (Pembrey et al. 2014). Transgenerational effects can arise in males or females and may be influenced by other environmental factors imposed on the progeny as well as genetic factors (Pembrey et al. 2014). Such observations highlight the importance of tracking and understanding the paternal effects of environmental factors.

**Figure 2:**
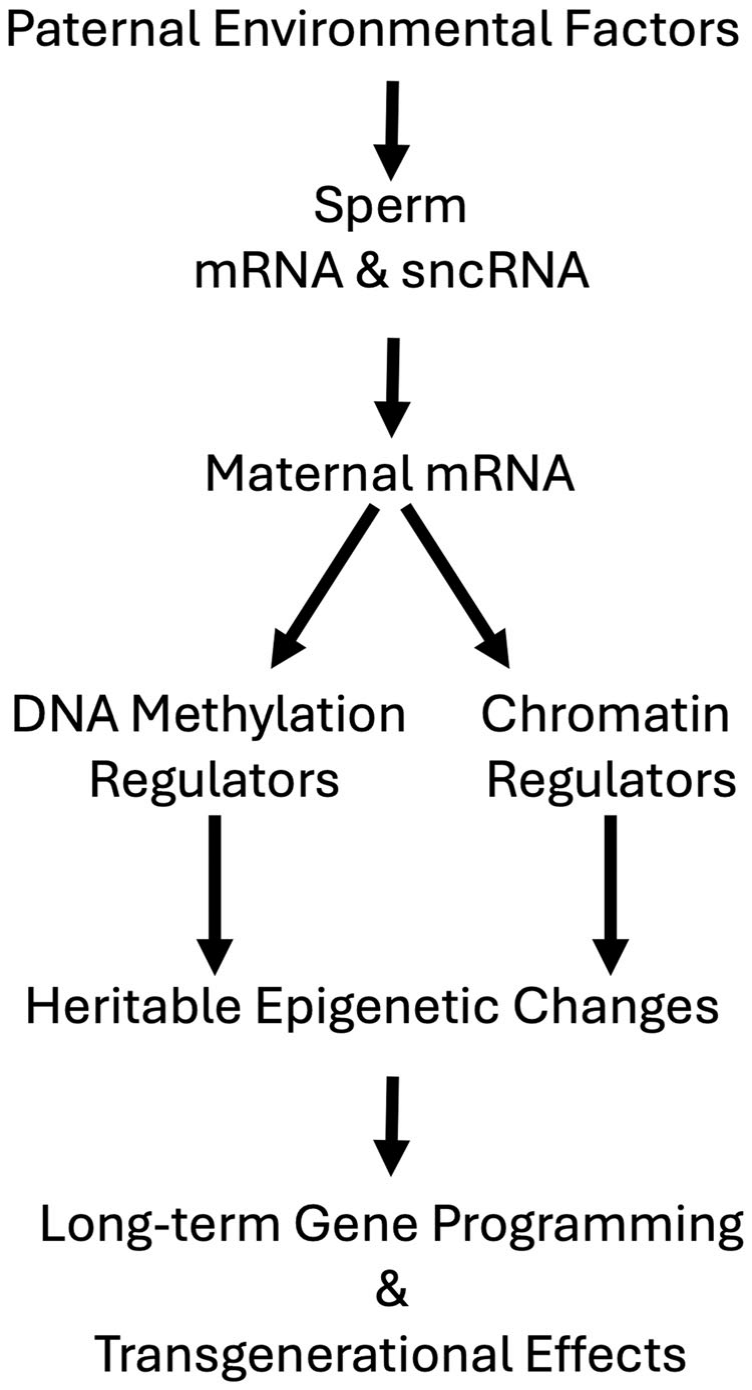
Proposed pathway from paternal stress to heritable epigenetic changes and transgenerational effects. Paternal environmental factors can include heat stress, oxidative stress, diet, toxin exposures, and other factors. These factors can alter the sperm content of macromolecular components such as mRNA and small noncoding RNA (sncRNA), which can, in turn, affect the translation and stability of maternal mRNAs. These maternal mRNA effects can result in altered expression of DNA methylation and chromatin regulators, leading to abnormal epigenetic modifications, paramutation, long‐term impacts on nuclear programming and development, and transgenerational phenotypic effects.

Many extrinsic or environmental factors can impact both sperm and semen quality and thereby generate or contribute to paternal effects on embryos and progeny, working through effects on maternal mRNA and other mechanisms discussed above (Figures 1 and 2). One of the major factors is heat. Heat stress in male mice can induce DNA damage and impair embryogenesis (Paul et al. 2008). Heat stress can also compromise sperm mitochondrial function (review, Abdelnour et al. 2022), increase reactive oxygen species (ROS) (Capela et al. 2022; Kujoana et al. 2024), induce germ cell apoptosis (Durairajanayagam et al. 2015), alter sperm mRNA and miRNA profiles (Celeghini et al. 2024), alter pronuclear size after fertilization (Rahman et al. 2018), and affect somatic cell function in the testis (Cai et al. 2021). Heat shock of bovine sperm reduces mitochondrial activity, increases ROS, increases caspase activity, and reduces fertilizing ability (da Silva et al. 2022). Additionally, bovine embryos fertilized with sperm subjected to progressively higher heat treatments display cleavage delay and preimplantation developmental arrest, along with an increased abundance in a small number of sperm miRNAs (da Silva et al. 2022). Similarly, summer heat stress in males can reduce sperm and semen quality, reduce embryo blastocyst formation, and diminish sperm motility in progeny bulls, exerting a transgenerational effect (Khan et al. 2023; Vanselow et al. 2024). And negative effects of high ambient temperature on human sperm were reported (Hoang‐Thi et al. 2022). Heat stress can impact spermatozoa derived from spermatids more severely than those derived from spermatocytes at the time of stress (Pérez‐Crespo et al. 2008). Additionally, heat stress of spermatozoa can lead to sex ratio distortion (Pérez‐Crespo et al. 2008). Collectively, these observations reveal a significant potential for paternal heat stress to impact embryos via alterations in sperm.

Oxidative stress can also impact sperm quality. Sperm cryopreservation can reduce glutathione concentration and increase ROS concentrations (Bollwein and Bittner 2018; Itahashi et al. 2022) and may exert diverse negative effects on sperm, including lipid peroxidation and attendant effects on membrane signaling, and mitochondrial electron transport defects (with further increases in ROS), sperm DNA damage, and protein oxidation (Bollwein and Bittner 2018). Addition of glutathione to sperm washing medium can enhance embryo development, although it is noted that glutathione can reduce ROS and also reduce disulfide bonds, thereby affecting protein structure with effects on DNA condensation (Itahashi et al. 2022). Oxidative stress associated with summer heat can affect sperm quality more highly in young versus older bulls (Bollwein and Bittner 2018). Additionally, embryos from patients with abnormal ovulation rates without polycystic ovarian syndrome displayed weak positive effects of sperm ROS on pronucleus formation and early cleavage, whereas embryos from normo‐ovulatory patients and patients with abnormal ovulation rates with polycystic ovarian syndrome displayed negative responses to sperm ROS (J. Liu, Zhu, et al. 2023). These observations indicate important interactions between sperm ROS, heat, male age, and oocyte factors.

An innovative method termed Sperm Energy Restriction and Recovery (SER) was developed in mice (Navarrete et al. 2019; Romarowski et al. 2023; Tourzani et al. 2022). This method is reported to improve sperm function, fertilization rate, blastocyst formation rate, and live birth rate, with an accompanying elevation in sperm Ca^2+^ concentrations (Tourzani et al. 2022). With this method, sperm are deprived of exogenous nutrients in vitro until motility diminishes, and then glucose alone or glucose and pyruvate are restored immediately before IVF (Romarowski et al. 2023; Tourzani et al. 2022). Sperm glycolysis, oxidative phosphorylation, and ATP content decline during the starvation period, and glucose alone is sufficient to induce an increase in glycolysis and oxidative phosphorylation (Romarowski et al. 2023). This SER treatment also alters global histone methylation and acetylation dynamics in both maternal and paternal pronuclei during the first cell cycle and in two‐cell stage embryonic nuclei, along with differences in embryonic gene expression at the two‐cell stage, including genes related to chromatin structure regulation (Tourzani et al. 2022). Interestingly, greater heterogeneity in histone modifications was seen for control embryos than SER‐derived embryos (Tourzani et al. 2022). It was speculated that the treatment might enhance embryo development by altering sperm miRNA and mRNA content or affecting sperm chromatin regions with bound transcription factors (Tourzani et al. 2022). These results suggest an important paternal effect connecting sperm energy dynamics and subsequent embryo phenotype.

Paternal diet and obesity also impact sperm function and subsequent embryo phenotype. Multiple mechanisms may mediate paternal obesity effects, and a recent review addressed many of these in detail, along with a consideration of confounding study variables that may impact reported effects or lack thereof (Peel et al. 2023). Combining clinical data with animal model studies provides striking evidence that paternal diet and obesity can mediate paternal effects in embryos and progeny via multiple effects on sperm. In humans, male obesity is associated with an increase in the proportion of sperm with reduced mitochondrial membrane potential, increased sperm DNA fragmentation, increased abnormal sperm morphology (Campbell et al. 2015), and reduced early embryo cleavage rate (Bibi et al. 2022). Associations of male obesity and high‐fat diet with increased sperm DNA fragmentation are prevalent in the literature, and it was noted that such effects can arise from multiple mechanisms (Peel et al. 2023). However, factors other than DNA fragmentation can contribute to effects on embryos and progeny. Using semen from bulls fed high gain diets for in vitro embryo production had no effect on sperm morphology or motility but resulted in reduced blastocyst formation rate (Seekford et al. 2023). Conversely, positive effects on blastocyst formation rate and reduced DNA fragmentation were reported for sperm obtained from bulls provided with an omega‐3‐enriched diet (Kalo et al. 2022). A male high‐fat diet in mice can alter gene methylation; the mouse SETD2 gene, for example, displays altered promoter DNA methylation (some sites increased, some reduced), and SETD2 expression increases in sperm of males fed a high‐fat diet, and progeny blastocysts display reduced total cell number and increased rates of apoptosis, and fewer live pups developed (Wei et al. 2023). A 2‐week high‐fat diet exposure increases the incidence of glucose intolerance in male progeny and alters the transcriptome in tissues such as muscle and adipose tissue, with effects on genes also associated with obesity in children (Tomar et al. 2024). Both low‐protein and high‐fat diet can diet affect small noncoding RNAs in sperm, and these effects are associated with diverse effects on progeny physiology and metabolism as well gene expression differences in preimplantation stage embryos (Klastrup et al. 2019). Importantly, about a quarter of sperm sncRNA abundances are sensitive to male high‐fat diet, with a reduction in nuclear tRNAs and an increase in mitochondrial tRNAs (Tomar et al. 2024). The mitochondrial tRNAs were transmitted and detectable in two‐cell stage mouse embryos, and this was associated with increased transcription of embryonic genes for oxidative phosphorylation, among other functions (Tomar et al. 2024). The mitochondrial tRNAs were also associated with BMI in human spermatozoa (Tomar et al. 2024). Male BMI affected 487 RNA elements in human sperm, including genes associated with CREWS (Swanson et al. 2020), and also affected sperm DNA methylation and specific sperm small noncoding RNAs (miRNAs, piRNAs, tRNA fragments) (Donkin et al. 2016).

The effects of male diet and obesity on sperm and subsequent preimplantation stage embryos extend to effects on progeny (Figure 2). For example, in humans, elevated paternal BMI and paternal obesity increase the risk of obesity in progeny in families with lean mothers (Tomar et al. 2024). Paternal obesity is associated with a reduced live birth rate and increased risk of pregnancy loss in studies of assisted reproduction outcomes (Campbell et al. 2015), as well as increased neonatal birth weight (Bibi et al. 2022). Additionally, mitochondrial tsRNA transmission from sperm of male mice fed high‐fat diets to embryos was associated with increased expression of genes related to mitochondrial metabolism in progeny muscle across two generations (Tomar et al. 2024). Paternal low‐fat diet in mice led to altered DNA methylation and expression of genes related to metabolism (Carone et al. 2010). These observations collectively point to a significant potential for paternal diet and obesity to exert long‐term, even intergenerational and trans‐generational metabolic programming of gene expression and mitochondrial activity in embryos and progeny.

Other environmental factors can exert effects on embryos and progeny via sperm. In cattle, exposure of sperm to bovine viral diarrhea virus before IVF was reported to infect embryos and compromise their health (Rahim‐Tayefeh et al. 2023). Bovine sperm sexing was reported to negatively affect cleavage and blastocyst formation (Steele et al. 2020). The effects of many environmental toxins and exposures have also been examined. Paternal smoking, which may be accompanied by additional relevant variables, had no clinical effect on preimplantation development, although the time to first cleavage was reduced (Frappier et al. 2022). Endocrine‐disrupting chemicals can exert significant and, in some cases, deleterious effects on embryos via sperm after paternal exposure. Exposure of mouse sperm to the pesticide atrazine at 1 μM concentration can delay cleavage without effect on the blastocyst rate (Komsky‐Elbaz et al. 2021). However, exposure of sperm to 1 μM atrazine led to changes in blastocyst gene expression (Komsky‐Elbaz et al. 2021). Male phthalate exposure assessed at the level of urinary concentration was associated with a reduction in the formation of high‐quality human blastocysts (Wu et al. 2017) and changes in the abundances of sperm mRNAs encoding chromatin regulators (CREWS), a function also affected by male obesity (Swanson et al. 2020; Swanson et al. 2023). Moreover, male phthalate exposure via pharmaceuticals affects a diverse range of sperm RNA elements and genomic repeat expression in a manner that varies with whether exposure was acute, chronic, increasing, or decreasing (Estill et al. 2019). The sncRNA content of SP EVs was also altered in association with urinary phthalate levels in men (Oluwayiose et al. 2023c). Experimental male phthalate exposure in mice does not affect major features of reproductive performance (Oluwayiose et al. 2021), but is deleterious to sperm acrosome reaction and capacitation (Khasin et al. 2020), increases sperm production of ROS (Khasin et al. 2020), and leads to changes in sperm and Day 7.5 embryonic and extraembryonic DNA methylation, as well as changes in gene expression related to multiple pathways including stem cell development and cardiogenesis, oxidative phosphorylation and other key signaling pathways (Oluwayiose et al. 2021). Paternal phthalate exposure can also exacerbate high‐fat diet‐induced metabolic changes such as insulin resistance and impaired insulin signaling in F1 and F2 progeny of treated males, sex‐specific effects in F2 females, and changes in sperm tsRNAs and rsRNAs (J. Liu, Shi, et al. 2023). Sperm exposure to 10 μM aflatoxin B in cattle alters the sperm proteome and exerts later effects on blastocyst expression of genes affecting developmental and other pathways (Komsky‐Elbaz et al. 2020). Treating mouse sperm with the pesticide bifenthrin from 0.1 to 100 μM reduced blastocyst formation in a dose‐dependent manner (Bae and Kwon 2021). Exposure of bovine sperm to another endocrine‐disrupting chemical, tributyltin (0.1 μM), reduces sperm motility and mitochondrial membrane potential and inhibits embryo cleavage and blastocyst formation (Daigneault and de Agostini Losano 2022). The fungicide vinclozolin has also been reported to affect sperm small noncoding RNA content (Schuster et al. 2016).

Collectively, these observations reveal significant impacts of a wide range of environmental factors on many of the sperm components and characteristics discussed earlier in this review, which can exert paternal effects on progeny, with significant effects observed at preimplantation embryonic or later stages, as well as some effects seen transgenerationally. Indeed, additive transgenerational effects being incurred across multiple generations of exposure (King and Skinner 2020; Nilsson et al. 2022, 2023) can allow individual toxins, combinations of toxins, or combinations of toxins with other environmental factors to exert diverse long‐term and transgenerational effects via epigenetic changes mediated by changes in DNA methylation, histone modifications, and sperm noncoding RNAs (McSwiggin et al. 2024). Thus, there is tremendous potential for diverse phenotypic effects in progeny mediated by a variety of factors that affect sperm components, particularly sncRNAs, contributing to a complex array of disease susceptibilities (Figure 2).

Different forms of stress can impact sperm quality and embryo phenotype. Paternal chronic hypoxia was reported to adversely affect embryo development in mice, along with changes in embryo gene methylation and gene expression (Tao et al. 2022). Physical exertion was noted as an important factor in a recent meta‐analysis (Giulioni et al. 2023). Long‐term infusion of male mice with the hormone ghrelin can inhibit preimplantation development (Belén Poretti et al. 2023). Restraint stress in mice was reported to retard growth and induce elevated serum glucose levels, lower anxiety and increase risk‐taking behaviors in progeny, negatively affect sperm characteristics in treated males and progeny numbers, and reduce DNA methylation at DMRs inherited intergenerationally (Zheng et al. 2021). Among those demethylation sites, 11.36% were observed transgenerationally, albeit with some alteration in re‐establishment proportion and methylation levels (Zheng et al. 2021). The DNA demethylation sites were associated with genes related to a set of signaling pathways, chromatin remodeling, and behavioral functions (Zheng et al. 2021). Effects on small sperm noncoding RNAs were also reported, with a bias toward overexpression of certain tsRNAs and underexpression of certain miRNAs (Zheng et al. 2021).

### 1.8 Impacts of Male Age on Sperm Quality and Fertility

Advancing male age is negatively associated with fertility, as it is accompanied by reduced semen volume, reduced sperm production and sperm count, reduced sperm motility, and changes in sperm function (Dong et al. 2022). As mentioned in several of the preceding sections, male age interacts with many of the intrinsic and extrinsic factors contributing to paternal effects via multiple mechanisms. Age effects include negative effects on DNA and epigenome integrity, an interaction with obesity, effects on mRNA and miRNA expression, and an interaction with oxidative stress. Interestingly, sperm telomere length increases with age and longer telomere length has been proposed as a marker of higher sperm quality (Dong et al. 2022; Laurentino et al. 2020; Rocca et al. 2019). This contrasts with telomere shortening observed in somatic cell types, which can be exacerbated by extrinsic factors (Rocca et al. 2019). The longer telomere length is associated with greater embryo viability (Rocca et al. 2019).

## 2 Conclusions and Perspectives

As our knowledge and understanding of sperm contributions to the early embryo increases, so too does our understanding of the mechanisms by which a variety of paternal effects are mediated. The potential for essential interactions between sperm factors and oocyte factors is striking, so that efforts to understand and treat infertility in many couples may require assessment of both male and female genetic, health, and environmental factors. Further study may ultimately reveal genetic factors that create quantitative differences in sperm and egg compatibility, reminiscent of extreme cases of incompatibility in mammals such as the *Ovum mutant* locus effect in mice (Baldacci et al. 1992; Bell et al. 2006; Hao et al. 2009; Pardo‐Manual de Villena et al. 1996, 1997, 1999; Song et al. 2011; Wakasugi 1973, 2007; Wakasugi et al. 1967). Further study may also provide a better understanding of the additive effects of prior or ongoing environmental exposures or health status of both male and female members of couples, alone or in combination with genetic factors. For example, prior exposure to factors that enhance ROS in both sperm and oocyte could exert additive effects on embryo viability, and maternal genetic factors that define the ability to respond to ROS could further contribute.

The extensive potential for impacts of diverse environmental factors on sperm, with attendant paternal effects on embryos and progeny, including inter‐ and transgenerational effects, could present a significant challenge to male fertility in both the human clinical setting and agricultural endeavors that rely on maximizing the viability and numbers of in vitro produced embryos. An increasing population age, declining birth rate, and many associated potential negative societal consequences in many developed countries have prompted consideration of possible approaches to reverse these trends, including the use of assisted reproduction technologies and increasing awareness of the negative effects of maternal and paternal age on reproduction (Bhasin et al. 2019; Hoorens et al. 2007; Ziebe and Devroey 2008). Aside from age, however, the impact of climate change, including heat stress, as well as environmental toxins and pollutants on human and livestock reproduction, has been recognized (Boekhorst et al. 2024; Lucy and Pohler 2025). Although broader societal consequences of changing human population demographics related to fertility have long been recognized (Hoorens et al. 2007; Ziebe and Devroey 2008), the discovery of inter‐ and transgenerational impacts of diverse environmental factors, including male exposures (Nilsson and Skinner 2015), presents an additional variable for human fertility, disease prevalence, and population dynamics. This should be addressed in models to assess the roles of historical, current, and future environmental exposures on human health, in models to address the value of assisted reproduction technologies in human fertility and total birth rate, and the potential role of assisted reproduction technologies in reducing the burden of certain diseases and disorders in the human population by mitigating effects of environmental exposures. Additionally, risks of assisted reproduction technologies introducing transgenerational effects should also be minimized.

Indeed, new pharmacological approaches to protect and improve sperm quality and mitigate the risks of adverse paternal effects on progeny are being actively studied. A recent study found that treatment of both mouse and human sperm in vitro with the compound BGP‐15 enhanced sperm quality (increased motility, increased mitochondrial membrane potential, reduced DNA oxidation, reduced DNA fragmentation) and improved early mouse embryo development (Gonzalez, McPherson, et al. 2024). BGP‐15 also mitigated the effects of aging on mouse sperm quality and embryo development (Gonzalez, Campugan, et al. 2024). BGP‐15 is a nicotinic amidoxime derivative originally studied as a drug to treat insulin resistance, and has diverse cellular effects, including inhibition of heat shock factor 1 acetylation, inhibition of Jun N terminal kinase (JNK) phosphorylation of the insulin receptor, inhibition of poly (adenosine 5′ diphosphate ribose) polymerase (PARP1), increase in AKT activity, activation of RAC1, and increase in membrane fluidity, through which it can increase heat shock protein activity, reduce ROS production, inhibit PARP activation, and reduce cell death (Pető et al. 2020). Additionally, coenzyme Q10, mitochondrial antioxidants (e.g., MitoQ), and other antioxidants have been widely studied in sperm washing media and semen extenders, as in vivo dietary supplements, and in conjunction with sperm cryopreservation to enhance sperm quality, with generally positive results (e.g., Allam et al. 2022; Dogan et al. 2024; Gonzalez et al. 2022; Hatami et al. 2024; Khazravi et al. 2024; Ma and Sun 2022; Masoudi et al. 2024). The increased understanding of the impacts of sperm factors (e.g., mRNAs and sncRNAs) on embryo viability and progeny phenotype provides a valuable basis for additional studies to assess the overall efficacy of such treatments in reducing the risks of effects of paternal exposures on progeny as well as inter‐ or transgenerational effects of such exposures.

## Author Contributions

**Keith E. Latham:** conceptualization, investigation, writing – original draft, writing – review and editing, project administration, resources, visualization.

## Ethics Statement

The author has nothing to report.

## Conflicts of Interest

The author declares no conflicts of interest.

## Data Availability

The data that support the findings of this study are available in Pubmed at https://pubmed.ncbi.nlm.nih.gov/. These data were derived from the following resources available in the public domain: Pubmed, https://pubmed.ncbi.nlm.nih.gov/.
